# Variations in Renal Tract Calculus Composition Between South Asian and Non-South Asian Ethnicities in North West England

**DOI:** 10.7759/cureus.97488

**Published:** 2025-11-22

**Authors:** John Gibson, Richard Simpson, Sarah Michael, Kirolos Michael, Adam Jones, Parthvi Vanalia, Max Mokete, Ehab Abusada, Raveendra Surange, Andreas Bourdoumis, Shalom Srirangam

**Affiliations:** 1 Urology, East Lancashire Hospitals NHS Trust, Blackburn, GBR; 2 Urology, Northern Care Alliance, Oldham, GBR; 3 Urology, Lancashire Teaching Foundation Trust, Preston, GBR; 4 Urology, Royal Oldham Hospital, Oldham, GBR

**Keywords:** ethnicity, renal calculi, south asian population, stone composition, urolithiasis epidemiology

## Abstract

Introduction

Ethnic variations in renal tract calculus composition remain poorly understood, despite an increasing global burden of urolithiasis. This study aimed to investigate differences in stone composition between South Asian and non-South Asian populations in North West England.

Methods

A retrospective cohort study was conducted using data from three NHS Hospital Trusts over five years (2015-2020). Stone composition analysis of 2,131 calculi from 1,890 patients categorised the data into five groups: calcium oxalate, mixed calcium oxalate and uric acid, uric acid, calcium phosphate and mixed calcium oxalate and calcium phosphate. Multivariate binary logistic regression assessed the relationship between ethnicity and stone composition.

Results

South Asian ethnicity was significantly associated with a higher likelihood of forming mixed calcium oxalate and uric acid calculi (odds ratio {OR}, 2.903; p = 0.017) and a decreased likelihood of calcium phosphate calculi (OR, 0.461; p = 0.025) compared to other ethnicities. Ethnicity was not significantly associated with other stone types. Additionally, male sex and increased age were significant predictors of specific stone compositions.

Conclusion

South Asian patients demonstrated distinct patterns of renal stone composition, being more likely to form mixed calcium oxalate and uric acid calculi and less likely to develop calcium phosphate stones. These findings indicate an association between ethnicity and stone composition but do not imply causation. Further prospective studies incorporating dietary, metabolic and biochemical data are warranted to clarify the mechanisms underlying these differences and their potential clinical relevance.

## Introduction

Renal tract calculi are a common and increasingly prevalent condition in the United Kingdom and globally [1-4]. This rising incidence places a growing strain on healthcare systems, with associated clinical and socioeconomic implications [5,6]. The majority of stones are calcium-based, predominantly calcium oxalate or calcium phosphate, accounting for 60%-80% of cases [3,5,7-9], while uric acid stones constitute around 10% [7]. Established risk factors include increasing age and male sex [8].

What remains less well-characterised is whether stone composition varies by ethnicity, particularly among South Asian populations (i.e. those of Indian, Pakistani, Sri Lankan and Bangladeshi origin). Previous studies have reported higher rates of urolithiasis in both South Asian countries and patients of South Asian ethnicity, but limited research has examined whether this extends to differences in stone composition [8,10-12].

Given the sizeable South Asian population in North West England and the limited data exploring ethnicity-specific patterns in calculus composition, this study sought to investigate potential associations between ethnicity and renal calculus composition within this population [13]. Using laboratory-confirmed stone analyses from three NHS Hospital Trusts, we performed a retrospective cohort study comparing South Asian to non-South Asian patients to determine whether ethnicity independently predicts specific stone types.

## Materials and methods

Data for this study were collected from three geographically linked NHS Hospital Trusts in North West England over a five-year period (November 2015 to December 2020). The three hospitals collectively serve a catchment population of approximately two million people. All urinary tract stones submitted for laboratory analysis during this period were retrospectively identified through electronic patient records and pathology databases. Stone composition was determined by quantitative Fourier-transform infrared (FTIR) spectroscopy performed at an independent biochemistry laboratory, ensuring standardised methodology and impartial results. All patients who had undergone urinary tract calculus composition analysis were included.

Demographic information recorded included patient age, sex and ethnicity; stone composition (expressed as the relative proportion of the principal calculus constituents); and, where available, serum urate and calcium levels. As the regional population is predominantly White British with a sizeable South Asian minority, South Asian patients were compared to a combined 'non-South Asian' group comprising all other ethnic backgrounds (including White British, Black, mixed and other categories) [13]. This pragmatic grouping reflected the demographic composition of the study region; allowed comparison between the South Asian population, the primary focus of the study, and all other ethnicities; and ensured adequate statistical power.

Stones in which a single component accounted for more than half of the total composition were classified according to that dominant constituent, whereas those with a secondary component exceeding 10% were categorised as mixed calculi. Patients with cystine and xanthine stones were excluded because of their rarity and well-established genetic predisposition. Likewise, stones composed of struvite, ammonium urate or calcium carbonate were excluded as they are commonly infection-related.

Following composition analysis, data were categorised into five principal groups based on chemical composition (see Appendices for full definitions): calcium oxalate, mixed calcium oxalate and uric acid, uric acid, calcium phosphate and mixed calcium oxalate and calcium phosphate calculi [14,15]. These categories represent the most common and metabolically distinct stone types encountered in clinical practice. The inclusion of the two mixed groups reflects the frequent overlap observed in stone analyses and shared metabolic and physicochemical pathways. This classification, consistent with prior literature, provides clinical relevance while maintaining adequate group sizes for statistical comparison [14,15].

Continuous variables were assessed for distribution using the Shapiro-Wilk test. Group differences for categorical data were examined with the chi-square test, and relationships between nonparametric variables were explored using the Spearman correlation. Variables demonstrating a p-value of <0.1 on univariate testing were subsequently entered into multivariable binary logistic regression models to estimate adjusted odds ratios (OR) with 95% confidence intervals (CI). Analyses were conducted on complete cases only; missing biochemical results were excluded and not imputed. IBM SPSS Statistics for Windows, version 25 (IBM Corp., Armonk, NY) was used for all analyses, and statistical significance was set at p < 0.05.

## Results

In total, stone composition data were available for 2,279 cases. After excluding 141 cases due to the presence of struvite, cysteine or xanthine stones, we analysed 2,131 calculus composition results from 1,890 patients. Among these patients, 366 (19.4%) were of South Asian origin, and 1,266 (67%) were men. The median age across all compositions was 47.4 years (range: 15-91 years); the highest mean age observed in uric acid stones was 64.5 years (range: 29-91 years), and the lowest in calcium phosphate stones was 50.0 years (range: 15-91 years). See Table 1 for these results.

**Table 1:** Demographics according to calculus type P-values were calculated using the chi-square (χ²) test, and p < 0.05 was considered statistically significant *Significant p-values Ca-Ox, calcium oxalate; UC, mixed uric acid/calcium oxalate; UA, anhydrous uric acid; Ca-Pho, calcium phosphate; Ca-Ox/Ca-Pho, mixed calcium oxalate/calcium phosphate

	Ca-OX	UC	UA	Ca-Pho	Ca-Ox/Ca-Pho	P (χ²)	χ²
	(n = 669)	(n = 108)	(n = 96)	(n = 572)	(n = 686)		
Sex							
Female	162	25	20	291	205	<0.001*	120.21
n (%)	24.20%	23.10%	20.80%	50.90%	29.90%		
Male	507	83	76	281	481		
n (%)	75.80%	76.90%	79.20%	49.10%	70.10%		
Ethnicity							
South Asian	155	30	11	77	140	<0.001*	28.16
n (%)	23.20%	27.80%	11.50%	13.50%	20.40%		
All other ethnicities	514	78	85	495	546		
n (%)	76.80%	72.20%	88.50%	86.50%	79.60%		
Other demographics							
Age, mean (range)	56.2 (16-89)	60.6 (24-90)	64.5 (29-90)	50.0 (15-91)	52.8 (19-90)	-	-
Serum calcium (mmol/L), mean (range)	2.4 (2.1-3.0)	2.4 (2.2-2.8)	2.3 (2.2-2.4)	2.4 (2.1-3.0)	2.4 (1.9-2.8)	-	-
Serum urate (mcmol/L), mean (range)	358.0 (191-676)	390.9 (237-519)	370.0 (275-514)	314.2 (191-539)	333.2 (143-527)	-	-

Ethnicity and calculus composition

The relationship between ethnicity and calculus composition was examined using multivariate binary logistic regression analyses. South Asian ethnicity was significantly associated with Group 2 calculi (mixed calcium oxalate and uric acid) (OR, 2.903; p = 0.017) and negatively associated with Group 4 calculi (calcium phosphate) (OR, 0.461; p = 0.025) compared to all other ethnicities. No statistically significant associations were found between ethnicity and Group 1 (calcium oxalate), Group 3 (uric acid) or Group 5 (mixed calcium oxalate and calcium phosphate) compositions. See Table 2 for these results in full.

**Table 2:** Multivariate logistic regression analysis for predictors of calculus composition P-values were derived from multivariate binary logistic regression using the Wald test, and p < 0.05 was considered statistically significant. Wald statistic values were not retained from the original analysis output *Significant values Ca-Ox, calcium oxalate; UC, mixed uric acid/calcium oxalate; UA, anhydrous uric acid; Ca-Pho, calcium phosphate; Ca-Ox/Ca-Pho, mixed calcium oxalate/calcium phosphate; OR, odds ratio; CI, confidence interval

	Ca-OX		UC		UA		Ca-Pho		Ca-Ox/Ca-Pho	
	OR (CI)	P	OR (CI)	P	OR (CI)	P	OR (CI)	P	OR (CI)	P
Demographic variable										
Age	1.009 (0.991-1.028)	0.335	1.049 (1.017-1.082)	0.002*	1.046 (0.971-1.127)	0.236	0.962 (0.942-0.982)	<0.001*	1.000 (0.982-1.018)	0.993
Sex (male versus female)	2.107 (1.128-3.936)	0.019*	0.458 (0.183-1.147)	0.096	1.384 (0.138-13.913)	0.783	0.247 (0.136-0.447)	<0.001*	2.889 (1.574-5.301)	0.001*
Ethnicity (South Asian versus Other)	1.050 (0.596-1.852)	0.865	2.903 (1.214-6.941)	0.017*	0.885 (0.089-8.762)	0.917	0.461 (0.234-0.908)	0.025*	1.127 (0.654-1.942)	0.667
Serum variable										
Calcium	0.493 (0.048-5.096)	0.553	0.072 (0.002-2.533)	0.148	0.155 (0.000-978.690)	0.677	36.642 (2.722-493.242)	0.007*	0.435 (0.042-4.544)	0.487
Uric acid	1.002 (0.999-1.005)	0.198	1.008 (1.003-1.013)	0.002*	1.002 (0.991-1.013)	0.742	0.997 (0.993-1.001)	0.158	0.996 (0.993-0.999)	0.019*

Sex, age and calculus composition

Further analysis revealed that male gender was a positive predictor for Group 1 (calcium oxalate) (OR, 2.107; p = 0.019) and Group 5 (mixed calcium oxalate/calcium phosphate) (OR, 2.889; p = 0.001) calculi but a negative predictor for Group 4 calculi (calcium phosphate) (OR, 0.247; p < 0.001). Increased age and higher serum uric acid levels were positive predictors for Group 2 calculi (mixed calcium oxalate and uric acid) (OR: 1.409 and p = 0.002; OR: 1.008 and p = 0.002, respectively). Conversely, increased age negatively predicted Group 4 calculi (calcium phosphate) (OR, 0.962; p < 0.001).

No statistically significant relationships were found between increased age and Group 1 (calcium oxalate), Group 3 (uric acid) or Group 5 (mixed calcium oxalate and calcium phosphate) compositions nor between male sex and Group 2 (mixed calcium oxalate and uric acid) or Group 3 (uric acid) compositions.

Overall calculus composition

Calcium-containing stones were the most prevalent, with 82.4% containing calcium in some form. Among the predefined subgroups, Group 5 (mixed calcium oxalate and calcium phosphate) was the most common (32.19%), followed by Group 1 (calcium oxalate, 31.39%) and Group 4 (calcium phosphate, 26.84%) calculi.

## Discussion

Our study of 2,131 calculi from an ethnically diverse area in England demonstrates variations in stone composition between ethnicities. South Asian ethnicity was associated with an increased prevalence of mixed calcium oxalate and uric acid calculi and a lower likelihood of calcium phosphate calculi. Our findings correspond with previous research showing that male sex and older age are linked with an increased risk of renal tract calculi and calcium oxalate calculi [16-19].

Research on renal tract calculus composition and ethnicity is limited. A smaller (n = 312) UK-based retrospective cohort study performed in 2017 by Vaggers et al. demonstrated no significant variations in calculus composition between ethnicities [20]. Similarly, Halinski et al. conducted a retrospective observational study across 10 countries (n = 710), evaluating differences in the composition of renal calculi, but did not use statistical tests to assess the significance of observed differences between populations [9]. Thus, our findings fill a notable gap in composition variations, particularly in individuals of South Asian ethnicity.

The specific composition of renal tract calculi informs diagnostic and management interventions. The European Association of Urology (EAU) guidelines support this, advising incorporating previous calculus analysis findings when planning treatment [21]. While our findings highlight differences in composition between ethnicities, these observations should be interpreted as associations rather than determinants of treatment. The study design does not allow conclusions about causality or direct clinical application, and further prospective research is required before any ethnicity-based prediction could inform management decisions.

Stone composition also provides insight into recurrence risk and underlying aetiology [22,23]. While adequate fluid intake is the mainstay of prevention, other dietary factors also play a role. The increased consumption of protein, calcium and salt correlates with higher urinary calcium levels and a consequent risk of calcium-containing calculus formation [24-26]. A diet high in animal proteins can predispose patients to uric acid calculi due to hyperuricosuria secondary to increased purine metabolism [26]. Although our study collected no dietary data, differences in habitual diet or metabolic profile between populations may partly underlie the observed associations. These factors warrant formal evaluation in prospective metabolic studies.

Given the recognised influence of diet on the risk of calculus formation, it is plausible that dietary acculturation among South Asian populations may contribute to the observed patterns. Traditional diets rich in fruits, vegetables and fibre are often replaced by more Westernised dietary habits characterised by the higher intake of animal protein, refined carbohydrates, processed foods, salt and sugar [27-29]. Such changes have been associated in previous studies with metabolic alterations, including hypercalciuria, hyperoxaluria, hyperuricosuria, aciduria and hypocitraturia, which may predispose individuals to mixed calcium oxalate and uric acid stone formation [26,30]. Moreover, higher sodium and sugar intake exacerbates calciuria and lower urinary pH [30]. These mechanisms remain hypothetical in our context but may provide a framework for future investigation. Likewise, the rising prevalence of type 2 diabetes among South Asian immigrants [28] is a known risk factor for uric acid calculi [31,32], which could contribute to the mixed calcium oxalate-uric acid profile observed in this study.

Research into ethnic disparities in urinary excretion has revealed that non-White patients generally have lower levels of urinary calcium, citrate and magnesium, key factors in stone formation and inhibition [33]. Similarly, research on South African populations shows that Black patients have higher urinary pH and oxalate levels but lower citrate and calcium excretion than White patients, yet paradoxically, they had a lower incidence of renal calculi [34]. These findings emphasise that differences in stone composition between ethnicities likely reflect a complex interplay of genetic, metabolic and environmental factors rather than ethnicity alone.

Although it is often assumed that South Asians have a higher incidence of uric acid stones, our study specifically found this association only with mixed calcium oxalate and uric acid stones. The reasons for this remain uncertain and merit further investigation. Future work should determine if these differences exist and uncover their underlying mechanisms, beyond standard risk factors, to include genetic and metabolic influences.

Our multi-centre, large-sample-size cohort from three NHS Trusts, located in a region with a higher-than-average South Asian population, enabled sufficient and meaningful ethnic comparisons. However, the retrospective design precluded adjustment for potential confounders, and only calculi submitted for analysis were captured, introducing the possibility of selection bias. Important clinical variables such as body mass index, diabetes, hypertension and other metabolic factors were not available in the dataset and therefore could not be included in the regression models; these should be incorporated in future prospective analyses to clarify the independent effect of ethnicity on stone composition. We lacked 24-hour urine data, which future prospective studies should include to link diet, metabolic excretion and stone type. We also did not stratify by calculus location nor differentiate within the heterogeneous 'South Asian' category; both warrant attention in subsequent research. Additionally, some mixed-stone subgroups contained smaller sample sizes, which may limit statistical power for detecting subtle associations. Finally, as our population is limited to North West England, generalisability beyond this region is uncertain.

## Conclusions

This multi-centre study identifies a novel association between South Asian ethnicity and specific renal stone compositions, with South Asian patients more likely to form mixed calcium oxalate and uric acid calculi and less likely to develop calcium phosphate stones. These findings align with established demographic influences such as age and sex and highlight the possible contribution of ethnicity in stone composition. The limited evidence in previous studies regarding ethnicity and calculus composition underscores the importance of our research. The study addresses a gap in current scientific literature, and our results suggest areas for further investigation and corroboration.

Despite our study's strengths, including a large dataset and multi-centre approach, limitations such as the retrospective nature and regional focus should be acknowledged. The associations observed may not imply causation, given the likely multifactorial determinants of calculus composition. Future research incorporating prospective data collection, detailed dietary and biochemical profiling and the stratification of South Asian subgroups will be essential to clarify the mechanisms underlying these associations.

## Appendices

Calcium oxalate calculi

This category includes both calcium oxalate monohydrate and dihydrate calculi, as differentiation between the two was limited by the lack of data. Calculi in this group did not have any minor constituent comprising more than 10% of the total composition.

Mixed calcium oxalate and uric acid calculi

Calculi primarily consisted of a combination of calcium oxalate and uric acid, with uric acid contributing to more than 10% of the total composition.

Uric acid calculi

Calculi are primarily composed of uric acid without the presence of calcium oxalate.

Calcium phosphate calculi

Calculi are composed predominantly of calcium phosphate.

Mixed calcium oxalate and calcium phosphate calculi

Calculi are composed of both calcium oxalate and calcium phosphate, where calcium phosphate makes up 10%-50% of the total composition.
